# Exploring patients’ experience and perception of being diagnosed with bladder cancer: a mixed‐methods approach

**DOI:** 10.1111/bju.15008

**Published:** 2020-02-12

**Authors:** Wei Shen Tan, Chin Hai Teo, Delcos Chan, Kar Mun Ang, Malgorzata Heinrich, Andrew Feber, Rachael Sarpong, Norman Williams, Chris Brew‐Graves, Chirk Jenn Ng, John Kelly, DETECT II trial collaborators, P. Khetrapal, A. Sridhar, H. Baker, F. Ocampo, N. Whotton, K. Dent, S. Pearson, J. Hatton, M. Newton, E. Heeney, K. Green, S. Evans, M. Rogers, A. Dann, J. Cook, M. Cornwell, R. Mills, H. Knight, S. Maher, A. Rane, S. Thomas, S. Reyner, G. Vallejera, P. Adeniran, S. Masood, S. Ridgway, M. Coulding, H. Savill, J. Mccormick, M. Clark, G. Collins, K. Jewers, S. Keith, G. Bowen, J. Hargreaves, K. Riley, S. Srirangam, R. Mistry, J. Chadwick, S. Cocks, R. Hull, A. Loftus, L. Dawson, H. Roberts, C. Main, S. Jain, C. Waymont, J. Rogers, A. Grant, V. Carter, H. Heap, C. Lomas, P. Cooke, Y. Baird, S. Moore, S. Greenslade, J. Margalef, I. Chadbourn, M. Harris, J. Hicks, P. Clitheroe, S. Connolly, S. Hodgkinson, H. Haydock, A. Sinclair, E. Storr, L. Cogley, S. Natale, W. Lovegrove, S. Smith, K. Smith, D. Hewitt, R. Sriram, K. Atkinson, L. Royle, J. Madine, K. MacLean, J. Walsh, A.M. Guerdette, M. Hill, D. Payne, A. Power, J. Cannon, L. Devereaux, A. Thompson, L. Scarratt, T. Hodgkiss, D. Johnstone, J. Johnson, J. Allsop, J. Rothwell, K. Connolly, J. Cherian, H. Wardle, D. Wilson, A. Bayles, S. Pelluri, J. Pati, A. Gherman, C. Scott, S. Madaan, J.A. Taylor, E. Edmunds, J. Moore, A. Rees, S. Williams, S. Caddy, S. Dukes, A. Goffe, K. Buckhorn, L. Nichols, P. Acher, K. Baillie, K. Middleton, C. Proctor, J. Cresswell, A. Chilvers, M. Cain, A. Vaux, D. Watson, S. Bradfield, H. Gregory, H. Mostafid, L. Kehoe, S. Drakeley, J.A. Davies, L. Williamson, R. Krishnan, N. Lunt, P. Hill, H. Burns, B. Townley, L. Wilkinson, H. Wassall, A. Sinclair, J. Hunt, B. Holbrook, L. Stancombe, J. Morrison, L. Vankoutrik, S. Misra, G. Fossey, A. Richards, K. Mcdonald, A. Henderson, R. Fennelly, M. Tribbeck, K. Ames, J. Borwell, M. Kotze, K. Beesley, K. Rennie, T. Porter, A. Gipson, L. Piper, L. Bailey, A. Chrisopoulou, K. Slevin, F. McCartin, H. Warburton, S. Hathaway‐Lees, K. Whetton, G. Delves, A. Day, T. Bankole, S. Broadhead, S. Malde, M. Oblak, D. Ellis, S. Bishara, T. Barias‐Lara, I. Donkov, H. Thatcher, H.M. Morris, C. Culmsee, A.H. Menzies, C. Bartlett, C. Cutting, N. O'Brien, R. Jannapureddy, A. Kelkar, J. Fitzgerald, S. Longhurst, C. Worth, A.M. Peracha, S. Mzazi, C. Poile, L. Griffiths, A. Cook, N. Barber, N. Brar, A. Alty, B. Zelhof, R. Blades

**Affiliations:** ^1^ Division of Surgery and Interventional Science University College London London UK; ^2^ Department of Urology Northwick Park Hospital London UK; ^3^ Department of Primary Care Medicine Faculty of Medicine University of Malaya Kuala Lumpur Malaysia; ^4^ Department of Medicine Royal Free Hospital London UK; ^5^ Department of Epidemiology and Public Health Health Behaviour Research Centre University College London London UK; ^6^ UCL Cancer Institute London UK; ^7^ Surgical & Interventional Trials Unit University College London London UK; ^8^ Department of Urology University College London Hospital London UK

**Keywords:** bladder cancer, diagnosis, interview, patient reported outcome measure, questionnaires, qualitative, #BladderCancer, #blcsm

## Abstract

**Objective:**

To determine patient experience and perception following a diagnosis of non‐muscle‐invasive bladder cancer (NMIBC).

**Patient and methods:**

Patients were part of a prospective multicentre observational study recruiting patients with NMIBC for a urine biomarker study (DETECT II; ClinicalTrials.gov: NCT02781428). A mixed‐methods approach comprising: (i) the Brief Illness Perception Questionnaire (Brief‐IPQ) and (ii) semi‐structured interviews to explore patients’ experience of having haematuria, and initial and subsequent experience with a NMIBC diagnosis. Both assessments were completed at 6 months after NMIBC diagnosis.

**Results:**

A total of 213 patients completed the Brief‐IPQ. Patients felt that they had minimal symptoms (median [interquartile range, IQR] score 2 [0–5]) and were not particularly affected emotionally (median [IQR] score 3 [1–6]) with a minimal effect to their daily life (median [IQR] score 2 [0–5]). However, they remained concerned about their cancer diagnosis (median [IQR] score 5 [3–8]) and felt that they had no personal control over the cancer (median [IQR] score 2 [2–5]) and believed that their illness would affect them for some time (median [IQR] score 6 [3–10]). A significant association with a lower personal control of the disease (*P* < 0.05) and a poorer understanding of the management of NMIBC (*P* < 0.05) was seen in patients aged >70 years. Many patients were uncertain about the cause of bladder cancer. Qualitative analysis found that at initial presentation of haematuria, most patients were not aware of the risk of bladder cancer. Patients were most anxious and psychologically affected between the interval of cystoscopy diagnosis and transurethral resection of bladder tumour (TURBT). Following TURBT, most patients were positive about their cancer prognosis.

**Conclusion:**

Patients with NMIBC have a poor perception of disease control and believe that their disease will continue over a prolonged period of time. This is particularly more pertinent in the elderly. Patients are most psychologically affected during the interval between cancer diagnosis following cystoscopy and TURBT. Health awareness about bladder cancer remained poor with a significant number of patients unaware of the causes of bladder cancer. Psychological support and prompt TURBT following bladder cancer diagnosis would help improve the mental health of patients with NMIBC.

**Abbreviations**
Brief‐IPQ: Brief Illness Perception Questionnaire
CIS: carcinoma *in situ*
EAU: European Association of Urology
EORTC QLQ: European Organisation for Research and Treatment of Cancer quality of life questionnaire
GCSE: general certificate of secondary education
IQR: interquartile range
MIBC: muscle‐invasive bladder cancer
NMIBC: non‐muscle‐invasive bladder cancer
(HR)QoL: (health‐related) quality of life
TURBT: transurethral resection of bladder tumour

## Introduction

Bladder cancer is the sixth most common cancer worldwide and is the ninth leading cause of cancer death 1. Most bladder cancers are diagnosed following a presentation of haematuria, with 75% of patients diagnosed with non‐muscle‐invasive bladder cancer (NMIBC) 2, 3. While NMIBC has a more favourable prognosis compared to muscle‐invasive bladder cancer (MIBC), up to a 50% of patients develop recurrence and 20% risk disease progression at 5 years 4.

Patients with NMIBC are initially treated with transurethral resection of bladder tumour (TURBT) and subsequently undergo repeated surveillance cystoscopy as often as every 3 months, with the aim of identifying recurrence early to allow for prompt treatment 5. These procedures are invasive and have the associated risk of bleeding, UTI, dysuria, and require attendance to hospital. Hence, this can have significant implications for the health‐related quality of life (HRQoL) of patients.

However, there remain limited reports of HRQoL in patients with NMIBC. Most reports focus on MIBC after radical cystectomy comparing outcomes following different urinary diversion techniques 6, 7. Meta‐analysis of patients after radical cystectomy suggest that HRQoL between continent and incontinent urinary diversion were comparable and patients take up to 12 months before HRQoL improves to baseline 8. In addition, there are no reports of qualitative assessment of patients’ experience following a diagnosis of bladder cancer.

In the present study, we used a mixed‐methods approach to determine patient’s experience and perception of being diagnosed with bladder cancer using semi‐structured interviews coupled with the Brief illness perception questionnaire (Brief‐IPQ), which uses a nine‐item scale designed to assess the cognitive and emotional representation of an illness 9. We hypothesised that patients with recurrent disease and those with higher‐risk disease were significantly more likely to have a worse HRQoL.

## Patients and methods

### Study design

To further explore patients’ experience of being diagnosed with bladder cancer, we utilised a mixed‐methods approach combining the Brief‐IPQ with qualitative semi‐structured interview. Patients completed the Brief‐IPQ at 6 months after a diagnosis of bladder cancer and subsequently, 20 consecutive patients (enriched for 20% G1 pTa NMIBC) who responded completed qualitative semi‐structured interviews or till saturation was reached. The time point of 6 months was deemed a suitable time point to determine patients’ experience of initial bladder cancer diagnosis and subsequent experience following early follow‐up. This mixed‐methods approach allowed for a more exploratory approach to triangulate and explain patients’ experience of a bladder cancer diagnosis.

Patients recruited were from a urine biomarker study (DETECT II; ClinicalTrials.gov: NCT02781428) recruiting patients following a new or recurrent diagnosis of bladder cancer. The full study protocol has been previously described 10, 11. The reported study represents a secondary endpoint for the DETECT II study.

### Participant eligibility, recruitment and data collection

A total of 370 patients with histologically confirmed NMIBC and ≥6‐months follow‐up were recruited from 52 UK hospitals between September 2016 and April 2017. The Brief‐IPQ was sent by post and 213 patients (57.6%) returned completed questionnaires (Figure S1). Subsequently, 20 English speaking patients from this cohort consented to participate in a semi‐structured telephone interview. All patients completed the interviews and Brief‐IPQ ≥6 months after a new NMIBC diagnosis.

Patient demographics, previous history of bladder cancer, complications after cystoscopy, as well as cancer stage, which was assessed using the WHO TNM cancer classification was recorded 12. European Association of Urology (EAU) risk classification was used to define bladder cancer risk 2.

### Brief‐IPQ

The Brief‐IPQ comprises nine questions 9. Five of the questions assess cognitive illness representations: (Q1) How much does your illness affect your life (consequences); (Q2) How long do you think your illness will continue (timeline); (Q3) How much control do you feel you have over your illness (personal control); (Q4) How much do you think your treatment can help your illness (treatment control); and (Q5) How much do you experience symptoms from your illness (identity). Two questions assess emotional representations: (Q6) How concerned are you about your illness (concern) and (Q8) How much does your illness affect you emotionally (emotional representation). One question assesses illness comprehensibility: (Q7) How well do you understand your illness (coherence). Each question has a Likert scale of 0–10. The final question (Q9) required patients to report their top three factors (in free text) that they believed caused their illness.

To compute the overall score, answer scales of three items [personal control (Q3), treatment control (Q4) and coherence (Q7)] were reversed and the sum for all eight questions were calculated, where a higher score (maximum score is 80) would imply a worse illness perception.

### Qualitative semi‐structured interview

All interviews were carried out by one interviewer (W.S.T.). The interviews were conducted via Skype (Microsoft, Redmond, WA, USA) and recorded using Evaer Video Recorder for Skype. The interview was designed to explore patients’ experience of being diagnosed with bladder cancer. This represents part of the interview to explore patients’ views and experience of using a non‐invasive urine biomarker test compared to cystoscopy, which has been previously published 10. The outline of the semi‐structured interview is shown in Table S1.

### Statistical methods and data analysis

Descriptive statistics such as mean, median, interquartile range (IQR) and 95% CI were used to report continuous data. Chi‐square was used to compare categorical variables, while the *t*‐test and anova were used for continuous variables. Factorial anova was used to determine categorical variables that were independently associated with a higher Brief‐IPQ score. Statistical analysis was performed using the Statistical Package for the Social Sciences (SPSS®), version 23 (SPSS Inc., IBM Corp., Armonk, NY, USA). Statistical significance was set at *P* < 0.05.

Interview recordings were transcribed, and data managed using Nvivo 11 (QSR International, Melbourne, Victoria, Australia). Open coding was performed by two researchers (W.S.T. and C.H.T.) on the first two transcripts and differences were resolved by discussion. A framework analysis was used to analyse the data as outlined: (i) Familiarisation – Transcripts of semi‐structured interview of participants were read; (ii) Indexing – Responses of participants were grouped according to the following themes based on patients’ illness trajectory: ‘Frist thoughts when experiencing haematuria’, ‘Patients’ experience of being diagnosed with bladder cancer’ and ‘Subsequent experience with bladder cancer diagnosis’; and (iii) Mapping and interpretation – Main descriptive comments were summarised according to the above themes. The remaining transcripts were then coded by one researcher (W.S.T.) and new emerging codes were discussed. To avoid potential bias in reporting, background notes throughout all study phases were reflected upon.

## Results

### Patient demographics

Baseline characteristics and clinicopathological variables for 213 patients who completed the Brief‐IPQ are reported in Table 1. The median patient age was 74.0 years and 63.4% (*n* = 135) of patients had a primary diagnosis of NMIBC. A total of 39.0% (*n* = 83) patients had high‐risk disease although only 17.8% (*n* = 38) of patients perceived their cancer as high risk.

**Table 1:** Patients’ demographics and clinicopathological variables.

Variable	Value
Number of patients	213
Age, years, median (IQR)	74.0 (67.1–81.1)
Gender, *n* (%)	
Male	170 (79.8)
Highest education, *n* (%)	
No formal education	8 (3.8)
High school	56 (26.3)
GCSE	39 (18.3)
A‐levels	20 (9.4)
University or higher degree	31 (14.6)
Not known	59 (27.7)
Smoking history, *n* (%)	
Non‐smoker	56 (26.3)
Ex‐smoker	129 (60.6)
Current smoker	18 (8.5)
Not known	10 (4.7)
Ethnicity, *n* (%)	
White	188 (88.3)
Non‐White	6 (2.8)
Not known	19 (8.9)
Employment, *n* (%)	
Full time/part‐time/home maker/voluntary	45 (21.1)
Retired	161 (75.6)
Disability/unemployed	4 (1.9)
Missing	3 (1.4)
New or recurrent tumour, *n* (%)	
New	135 (63.4)
Recurrence	78 (36.6)
Previous cystoscopies, *n* (%)	
≤2	66 (31.0)
2–5	92 (43.2)
≥6	47 (22.1)
Not known	8 (3.8)
Tumour grade, *n* (%)	
G1	36 (16.9)
G2	99 (46.5)
G3	71 (33.3)
Not known	7 (3.3)
Tumour stage, *n* (%)	
CIS	3 (1.4)
pTa	156 (73.2)
pT1	47 (22.1)
Not known	7 (3.3)
Papillary with concurrent CIS, *n* (%)	5 (2.4)
Disease risk, *n* (%)	
Low	18 (8.5)
Intermediate	105 (49.3)
High	83 (39.0)
Not known	7 (3.3)
Patients’ perception of disease risk, *n* (%)	
Low	49 (23.0)
Intermediate	112 (52.6)
High	38 (17.8)
Not known	14 (6.6)

CIS, carcinoma *in situ*; GCSE, general certificate of secondary education.

### Brief‐IPQ score

Overall, patients with NMIBC appeared to be coping well following a diagnosis of NMIBC with a median (IQR) Brief‐IPQ score of 32 (22–40). At 6 months after a diagnosis of NMIBC, patients reported that the disease had a minimal effect on their life (Q1) (median [IQR] score 2 [0–5]). Patients also reported minimal symptoms due to the cancer (Q5) (median [IQR] score 2 [0–5]) and that they were not particularly affected emotionally (Q8) (median [IQR] score 3 [1–6]) (Table 2). Patients were very optimistic that their treatment could control the disease (Q4) (median [IQR] score 9 [7–10]) and they acknowledged that they have a good understanding of the disease (Q7) (median [IQR] score 8 [6–10]). However, they remained moderately concerned about bladder cancer (Q6) (median [IQR] score 5 [3–8]). Most patients did not feel that they had personal control over their bladder cancer (Q3) (median [IQR] score 2 [2–5]) and believed that their illness would affect them for some time (Q2) (median [IQR] score 6 [3–10]).

**Table 2:** Brief‐IPQ scores.

Component	Score, median (IQR) [range]
1. Consequence (*n* = 205)	2 (0–5)
2. Timeline (*n* = 193)	6 (3–10)
3. Personal control (*n* = 202)	2 (2–5)
4. Treatment control (*n* = 199)	9 (7–10)
5. Identity (*n* = 202)	2 (0–5)
6. Concern (*n* = 201)	5 (3–8)
7. Coherence (*n* = 204)	8 (6–10)
8. Emotional representation (*n* = 202)	3 (1–6)
Overall score (*n* = 184)	32 (22–40) [0–65]

Table 3 reports the top three reasons patients perceived to be the cause for their bladder cancer diagnosis as part of the Brief‐IPQ. These included: not certain, smoking, lifestyle/diet, work environment/chemical exposure, increasing age, and family history/genetics.

**Table 3:** Patient’s perception on the top three reasons they developed bladder cancer (Brief‐IPQ question 9).

Rank	1st most important factor (*n* = 173)	2nd most important factor (*n* = 91)	3rd most important factor (*n* = 68)
1	Uncertain (*n* = 55)	Lifestyle/diet (*n* = 21)	Uncertain (*n* = 12)
2	Smoking (*n* = 36)	Smoking/passive smoking (*n* = 11)	Lifestyle/diet (*n* = 11)
3	Work environment/chemical exposure (*n* = 19)	Uncertain (*n* = 11)	Work environment/chemical exposure (*n* = 8)
4	Age (*n* = 15)	Family history/genetics (*n* = 6)	Family history/genetics (*n* = 6)
5	Family history/genetics (*n* = 8)	Stress (*n* = 6)	Bad luck (*n* = 4)
			Smoking (*n* = 4)

The overall Brief‐IPQ score between patients with a new bladder cancer diagnosis was similar to those with a previous diagnosis of bladder cancer (31 vs 34, *P* = 0.121) (Table S2). However, patients with a previous diagnosis of bladder cancer were significantly more likely to feel that their illness would continue (Q2) (7.5 vs 5.3, *P* < 0.001) and less likely to feel they have control over their disease (Q3) (2.4 vs 3.3, *P* = 0.046). Further, sensitivity analysis of Brief‐IPQ between patients treated with adjuvant intravesical treatment and those without adjuvant treatment did not show a difference in the overall and subcomponents of the Brief‐IPQ (all *P* > 0.05), except that patients treated with adjuvant intravesical treatment felt that their NMIBC had a more significant impact on their life (Q1) (3.4 vs 2.5, *P* = 0.027).

Correlation between patient demographics and clinicopathological variables stratified according to Brief‐IPQ scores is shown in Table 4. Younger patients believed they had a better control of their illness and understood their illness better compared to older patients (*P* = 0.035). Patients with disease recurrence were more likely to believe their disease would affect them over a prolonged period of time compared to patients with a new diagnosis (*P* < 0.001). Higher grade (consequence [*P* = 0.004], identify [*P* = 0.03]), stage (consequence [*P* < 0.001], timeline [*P* = 0.015], identify [*P* = 0.002], concern [*P* = 0.005], emotion [*P* = 0.009]), and disease risk (consequence [*P *< 0.001], timeline [*P* = 0.003], identity [*P* = 0.006], emotion [*P* = 0.038]) were significantly associated with a more detrimental effect to patients’ HRQoL. Patients’ perception of the risk of disease recurrence affected them cognitively and emotionally significantly more than the actual disease risk itself.

**Table 4:** Patients’ demographics and clinicopathological variables stratified according Brief‐IPQ scores.

	Brief‐IPQ score, mean (SD)								
Variables	Q1 Consequence	Q2 Timeline	Q3 Personal control	Q4 Treatment control	Q5 Identity	Q6 Concern	Q7 Comprehensibility	Q8 Emotion	Overall score
Age, years									
≤70	3.0 (2.5)	5.8 (3.5)	3.7 (3.1)	8.1 (2.5)	2.9 (2.8)	6.0 (3.1)	8.2 (2.1)	4.5 (3.1)	32.4 (12.5)
>70	2.8 (2.6)	6.2 (3.5)	2.7 (3.5)*	8.3 (2.5)	2.5 (2.7)	5.4 (3.3)	7.4 (2.9)*	3.5 (3.0)*	31.7 (12.2)
Gender									
Male	2.9 (2.6)	6.2 (3.5)	3.2 (3.3)*	8.3 (2.3)	2.6 (2.8)	5.4 (3.3)	7.6 (2.6)	3.5 (3.1)	31.5 (12.4)
Female	3.0 (2.6)	5.4 (3.7)	2.2 (2.5)	7.8 (3.1)	2.8 (2.7)	6.3 (3.1)	7.8 (2.8)	4.9 (3.1)	34.2 (11.6)
Highest education									
No formal education/GCSE	3.0 (2.7)	6.5 (3.5)	2.7 (2.8)	8.2 (2.5)	2.8 (2.8)	5.8 (3.3)	7.6 (3.3)	4.1 (3.0)	33.8 (12.1)
A levels/degree holder	2.8 (2.5)	5.7 (3.4)	3.0 (3.0)	8.4 (2.1)	2.4 (2.6)	5.6 (3.2)	8.1 (2.0)	3.8 (2.3)	30.6 (10.8)
Previous cystoscopies									
≤2	2.3 (2.2)	4.8 (3.5)	3.2 (3.3)*	8.0 (2.8)	2.0 (2.6)	5.1 (3.0)	7.6 (2.8)	3.4 (3.0)	28.8 (11.4)
2–5	3.3 (2.7)	5.9 (3.5)	3.3 (3.0)	8.5 (2.1)	2.9 (2.8)	6.0 (3.1)	7.4 (2.6)	4.1 (3.2)	32.7 (12.6)*
≥6	3.2 (2.8)	8.4 (2.3)**	1.9 (3.0)	8.0 (2.8)	3.1 (2.8)	5.5 (3.7)	8.2 (2.7)	3.8 (3.3)	36.4 (11.8)
New or recurrent cancer									
New	2.8 (2.5)	5.3 (3.5)	3.3 (3.1)	8.2 (2.4)	2.5 (2.7)	5.7 (3.1)	7.5 (2.6)	4.0 (3.0)	31.0 (12.4)
Recurrent	3.0 (2.7)	7.5 (3.1)**	2.4 (3.2)	8.2 (2.8)	2.7 (2.8)	5.4 (3.6)	8.0 (2.7)	3.4 (3.3)	34.0 (11.8)
Tumour grade									
G1	2.5 (2.3)	5.2 (3.6)	2.4 (2.9)	8.5 (2.2)	2.3 (2.6)	5.4 (3.2)	7.7 (2.9)	3.7 (3.2)	30.5 (11.6)
G2	2.6 (2.5)	6.1 (3.7)	3.3 (3.5)	8.3 (2.7)	2.4 (2.7)	5.3 (3.4)	7.8 (2.9)	3.3 (3.3)	29.9 (11.9)
G3	3.7 (2.7)**	6.3 (3.2)	3.2 (2.7)	7.9 (2.5)	3.4 (2.8)*	6.2 (3.0)	7.5 (2.2)	4.5 (4.5)	35.4 (12.9)*
Tumour stage									
Isolated CIS/pTa	2.6 (2.5)	5.7 (3.6)	3.3 (3.3)	8.4 (2.4)	2.4 (2.6)	5.3 (3.4)	7.8 (2.7)	3.4 (3.1)	29.7 (11.6)
pT1	4.2 (2.6)**	6.9 (3.1)*	2.4 (2.3)	7.6 (2.8)	3.9 (3.1)**	7.0 (2.4)**	7.1 (2.4)	5.1 (2.9)**	39.9 (11.9)**
Actual disease risk									
Low	2.2 (2.2)	3.7 (3.4)	2.1 (2.9)	8.6 (1.9)	1.7 (2.5)	5.7 (3.2)	8.2 (2.6)	3.4 (3.0)	27.9 (12.7)
Intermediate	2.4 (2.5)	6.1 (3.7)	3.4 (3.5)	8.4 (2.6)	2.3 (2.6)	5.1 (3.4)	7.7 (3.0)	3.2 (3.0)	29.4 (11.6)**
High	3.8 (2.6)**	6.4 (3.1)**	3.0 (2.7)	7.9 (2.5)	3.4 (2.8)**	6.3 (2.9)	7.5 (2.2)	4.6 (3.1)*	36.0 (12.3)
Patient perception of risk of recurrence									
Low	1.9 (2.3)	4.5 (4.0)	3.7 (3.8)	7.8 (3.3)	1.6 (2.6)	3.6 (3.2)	7.5 (3.3)	2.2 (2.7)	24.3 (11.2)
Intermediate	2.8 (2.4)	6.2 (3.2)	2.9 (2.9)	8.3 (2.1)	2.8 (2.7)	6.1 (2.8)	7.5 (2.5)	4.2 (3.0)	33.2 (11.1)
High	4.5 (3.0)**	8.2 (2.8)**	2.5 (2.9)	8.5 (2.7)	3.6 (3.0)**	6.9 (3.5)**	8.8 (1.8)*	4.7 (3.6)**	38.5 (12.5)**
Any adverse event									
Yes	3.0 (2.7)	6.3 (3.4)	3.2 (3.2)	8.4 (2.4)	2.9 (2.8)*	5.7 (3.3)	7.9 (2.6)	3.8 (3.2)	32.4 (12.7)
No	2.4 (2.2)	5.1 (3.7)	2.5 (3.0)	7.6 (2.8)	1.8 (2.5)	5.3 (3.0)	7.1 (3.0)	3.6 (2.8)	30.2 (10.3)
Haematuria									
Yes	3.4 (2.7)*	6.9 (3.2)*	3.3 (3.2)	8.5 (2.3)	3.1 (2.8)	5.9 (3.2)	8.0 (2.2)	4.0 (3.2)	33.3 (12.3)
No	2.5 (2.4)	5.7 (3.6)	2.5 (3.1)	7.9 (2.6)	2.3 (2.7)	5.3 (3.2)	7.4 (3.0)	3.7 (2.9)	31.4 (12.2)
Dysuria/LUTS									
Yes	3.0 (2.6)	6.5 (3.4)*	3.2 (3.1)	8.3 (2.4)	2.9 (2.7)	5.7 (3.3)	7.8 (2.5)	3.9 (3.2)	32.6 (12.1)
No	2.6 (2.7)	5.2 (3.7)	2.6 (3.2)	8.0 (2.8)	2.1 (2.8)	5.2 (3.1)	7.4 (2.9)	3.6 (3.0)	30.6 (12.4)
UTI requiring antibiotics									
Yes	3.6 (2.9)	6.5 (3.3)	3.2 (3.4)	8.2 (3.0)	3.6 (3.1)*	5.9 (3.4)	8.0 (2.5)	4.3 (3.5)	33.5 (15.9)
No	2.7 (2.5)	6.1 (3.6)	2.9 (3.1)	8.3 (2.3)	2.4 (2.6)	5.4 (3.2)	7.6 (2.7)	3.6 (3.0)	31.6 (10.9)

CIS, carcinoma *in situ*; GCSE, general certificate of secondary education.

* *P* < 0.05, **P < 0.01.

Factorial anova determined that higher tumour stage (*P *= 0.003) and patient perception of disease risk (*P* = 0.001) were significantly associated with poorer HRQoL based on the Brief‐IPQ independent of age, gender, educational level attained, number of previous cystoscopies, new or recurrent disease, tumour grade, tumour actual disease risk, and adverse events after cystoscopy.

### Qualitative analysis

The demographics and tumour characteristics of the 20 patients interviewed are shown in Table S3. The findings were categorised into three themes: (i) First thoughts when experiencing haematuria, (ii) Patients’ experience when being diagnosed with bladder cancer and (iii) Subsequent experience with bladder cancer diagnosis. No new themes emerged during the analysis. The quotations supporting the themes are described in Table 5.

**Table 5:** Qualitative analysis for advantages and disadvantages of cystoscopy ‐ excerpt from patient interviews.

**First thoughts when experiencing haematuria**
I had previously had a urine infection, which generated the same symptoms. I thought that this time it’s different and I went straight to my GP. He’s obviously prescribed something to tackle the UTI but felt I should be referred for further investigation. I wasn’t sure what to think really. I just knew it wasn’t normal. I knew something was up and because of previous blood in my urine, even though the blood in my urine previously wasn’t as heavy as this one, the one that brought me to the attention to the hospital. I didn’t think it was cancer. I thought maybe a prostate problem. I thought it might be some kind of prostate problem and also some kind of STD… some kind of kidney problem. Because of the blood in my urine. I never… cancer was the last thing I would have thought of… Well I remember vividly because I had seen the adverts on television or in paper you know “if there was blood in your pee go to your Dr” … I woke up and went to the toilet, and I wouldn’t say it was blood but it looked just like diarrhoea that it was passing. So I shouted to wife, you better go and have a look at this. She got a glass and I weed into that and it was just like a very thin diarrhoea. So she went off to the doctor got a sample bottle of all the proper stuff and made an appointment for me the following morning. In fact I had gone for blood test and I had blood in the urine…I thought the investigation would be for my prostate. I thought there was a chance that there might be prostate cancer

**Patients’ experience of being diagnosed with bladder cancer**
So I suppose after the first cystoscopy all I knew was that there was a growth. That was a worry. So it was…until they’d been a biopsy and no one could tell me where it was, you know what grade of tumour it was basically. There was a lot of waiting a long time between the flexi cystoscopy and then the operation. I was a bit shell shocked at the time. When I was first diagnosed, I felt terribly sick at the time because all on my own and I had no idea how severe or how bad it was as they have not done tests like the biopsy. I had no idea. It was pretty terrifying… I was petrified. The first thing that goes through your mind is how long… is it bad. It was just a jumble. My mind was just swirling around. I rang my wife, she then came to the hospital. I sort of calmed down a bit and we took it from there. Once I had the operation I spoke to the consultant and the urologists and everyone that have done it and I felt a lot better. I feel a lot calmer now. A bit disturbed and at that stage I didn’t know how severe the condition can be and yeah there is a certain amount of apprehension. I didn’t sort tremor in horror. Yeah. These things come to you and those are the cards that have been dealt and you have to deal with them. On the bed with the flexible cystoscopy and there was a screen and they said ‘oh there is a tumour’ or something like that. It wasn’t the best way to find out. Yes. My emotional it’s affected my emotional well‐being principally worry and anxiety. As soon as I got the diagnosis. I looked to see the kind of percentages of life expectancy in different scenarios. Well I think it kicks you in the teeth for a little bit but you know, No, I just took the first lot of treatment and I was given the all clear and I just assumed I would be kept an eye on for a couple years, every 3 months or something and then but obviously it hasn’t got that far. You know… I mean there’s no point in worrying for nothing. Well, I would say you know you get into that beforehand for which you have to go, is not a pleasant experience. So you try not to think about it though as it gets near you just create a level of anxiety. Mentally… not physically. I think what it was because my sister had bladder cancer but hers was so different to mine. She only had one course of chemotherapy with the liquid inside the bladder and then that was it. So naturally I thought I’d be same at those line. But I wasn’t and I had a lot more treatment than what she had. And I think the main thing that’s always in the back of my mind is will it ever be cured which it won’t be. A little bit upset. I already had some precognition that there was something serious in that in that area but it wasn’t the shock to me. So yeah. The chap who did it, the registrar or consultant that did, you know the matter of fact you’ve got cancer, we can operate but obviously I have had the CT scan to confirm that it hadn’t gone through the bladder wall, so that alleviated a lot of the concern that I have, because that it could be treated as well. Well I don’t know. I mean obviously you think about death. I mean I’ve got children, I was thinking about them but then after a while I just well I just start to come to realization, well, it seems to be it will be. I wasn’t worried as such. I had a belief that I wouldn’t succumb as in you know it wouldn’t be fatal for me, but then I had to be that kind of positive thing. I had to have that positive thinking. I didn’t really tell my kids too much. My sister pretty sympathetic, my sister was pretty helpful. I’ve got cancer. Am I going to die? You know I mean 50 years ago I probably would have done but I’m hoping I might just have another year or two… No it just means what we always say or be like you know just get over that and then you walk under a bus and it’s all over. At first it was shock and disbelief but it was quite, very psychologically. I mean I overcame that… quite rapidly to be fair because I was able to sort of digest it and said look, you know I need to move on and I need to see what is going next. Plus it was only the diagnosis part at the moment. I still had to go through the treatment and you know there’s still a lot to look for. Well quite shocked because I didn’t know much about it… Erm…I was like okay, not too worried about it. I saw it as a minor thing, they will operate on and take it out, and hopefully all will be alright after that. Uh… Well it didn’t really worry me actually. They just say you know it has been caught early so it shouldn’t be any problem. The tumour is related to smoking but I don’t smoke so its bad luck You know it could have been could have been a lot worse that I’m lucky that it was found early. They think that amongst the type of cancers. I shouldn’t have a recurrence.

**Subsequent experience with bladder cancer diagnosis**
No not really. I’m fairly sanguine about it. No I’m very positive. Now that I have had two cystoscopies and I am not due for another 6 months back in August, I’ve got a handle on it. I understand what it is. I got back my normal daily life and gone back to work. I haven’t got time to sit down worrying about it, if it’s there, it’s there, if it’s not, it’s not you know what I mean? Now I feel fine and the catheterisation and insertion of the BCG doesn’t you know it’s not really a big problem at all. It doesn’t cause discomfort or anything like that. No basically. It’s okay. It hasn’t affected me. No, no. In fact I wasn’t ill beforehand. Obviously, no symptoms no fear. Apart from I did have a slight discomfort when I was walking. Technically I haven’t been ill. It’s not obviously affecting any diet or anything I didn’t feel any different physically than I did prior to finding out that I had the cancer so… There was no change in my in my life physically, mentally obviously it affects to a certain extent…but I feel pretty okay at least. Sometimes I think about it but it doesn’t stick to my mind. A month ago, I had an examination to see if it has come back and they said it was all clear, I’ve got another examination in August … So I’ve tried to adjust my diet to eat more healthily… You know I was affected when I had to have a catheter when I came out of hospital. Other than that no… I’m watching my diet. I am drinking a lot of fluids and I’m avoiding caffeine alcohol. Yeah because really, I never had any symptoms

#### First thoughts when experiencing haematuria

All patients were aware that haematuria was not physiological. The ‘blood in pee’ campaign increased patient awareness about the dangers of haematuria and triggered patients to visit their primary care doctor for further evaluation. However, most did not suspect that bladder cancer could be responsible for haematuria. UTI, sexually transmitted disease, kidney and prostatic problems were the patients’ initial suspected causes of their haematuria.

#### Patients’ experience when being diagnosed with bladder cancer

Patients were worried and anxious when diagnosed with bladder cancer after flexible cystoscopy. Some patients felt that their cancer diagnosis was due to bad luck. While they were not affected physically and remained asymptomatic, they were clearly psychologically affected. Most patients were worried about the threat bladder cancer had to their life. Patients found family support and the ability to discuss their diagnosis with their family helpful psychologically. The uncertainty about diagnosis makes patients anxious and this persists until TURBT histology has confirmed the diagnosis and cancer prognosis was discussed. A minority of patients faced the diagnosis of bladder cancer with a calm demeanour. While they were concerned, they were aware that worrying about cancer prognosis was not helpful and patients had a positive outlook prior to TURBT.

#### Subsequent experience with bladder cancer diagnosis

Following TURBT, most patients were positive about their cancer prognosis. They remained asymptomatic and bladder cancer did not affect their HRQoL. They understood the recommendations for repeated cystoscopies and did not find this prohibitive. Patients who required intravesical treatment tolerated this well. Some patients had made lifestyle changes such as changes to their diet after their bladder cancer diagnosis.

## Discussion

We report the first study to use a mixed‐methods approach to evaluate patients’ experience of being diagnosed with NMIBC. We utilised the Brief‐IPQ to assess this for the first time in patients with NMIBC. This generic HRQoL tool was developed to allow comparison between different cancers. The Brief‐IPQ was coupled with a qualitative semi‐structured interview to explore patients’ perception after a bladder cancer diagnosis in more detail.

Regular cystoscopies, which can be lifelong in high‐risk NMIBC, are likely responsible for patients’ perception that their cancer management will continue. This in turn, makes patients anxious and feel a lack of control over their disease. Our present findings are consistent with a mix‐methods study by Ranchor et al. 13 which reported that poor perception of disease control was associated with higher psychological distress. Indeed, others have also reported that at 6‐months follow‐up patient’s mental health remains significantly worse compared to baseline 14. Specifically, perception of poorer control over their illness was significantly associated with older patients (>70 years). We hypothesised that this may be reflected by their poorer understanding of bladder cancer. This represents an area of need where patients with NMIBC may benefit from psychological support, especially early on in their diagnosis, and older patients may require additional patient education and support with regards to understanding better the management of their disease. The fact that a significant number of patients were uncertain about what led to their bladder cancer diagnosis, suggests that an increase in public health awareness is necessary. Only 20.8% of patients who responded attributed smoking as the leading cause for their bladder cancer diagnosis.

Qualitative analysis suggested that at initial diagnosis, most patients felt physically well. This was not unexpected, as most patients were diagnosed following a presentation of haematuria and were otherwise asymptomatic 3. Nevertheless, patients were psychologically affected particularly between the period of initial cancer diagnosis after flexible cystoscopy and TURBT. This interval represents a period of great uncertainty, where patients are often told of a diagnosis of cancer given the high positive predictive value of cystoscopy but are not informed of disease prognosis given the lack of histopathology 15. While the implementation of the ‘2‐week wait’ pathway in the UK has reduced the time from primary care referral to urology consultation for the investigation of haematuria and diagnosis of bladder cancer, the time to treatment has not reduced 16. After TURBT, most patients had a positive outlook of their illness and interestingly, some had adopted a healthy lifestyle since their cancer diagnosis.

When compared to patients with a diagnosis of endometrial, colorectal, non‐Hodgkin lymphoma and myeloma, patients with NMIBC report better cognitive ability and emotional states 17. Patients with NMIBC were less affected by their cancer, were more optimistic about their treatment, reported fewer symptoms and had a better understanding of their illness. However, patients with NMIBC reported that their disease was more likely to continue, which may be due to the fact that all patients were assessed ‘soon’ after a diagnosis of bladder cancer (6 months) and that some patients with bladder cancer will require life‐long cystoscopy surveillance. Similar to other reports, patients with more adverse oncological features were more likely to be concerned about their cancer; this affected them emotionally and in their overall life 17.

A recent systematic review of HRQoL studies in patients with NMIBC analysed 14 quantitative studies and one mixed‐methods study, of which 11 studies had only patients with NMIBC. The most commonly used questionnaire was the European Organisation for Research and Treatment of Cancer quality of life questionnaire 30‐item core (EORTC QLQ‐C30; five studies) and the EORTC QLQ NMIBC24 (three studies) 18. The studies included were heterogeneous with sample sizes of between 30–244 patients. Six studies compared HRQoL of patients after treatment with different adjuvant intravesical chemotherapy protocols, suggesting that intravesical instillation affects overall HRQoL 19, 20, 21, 22, 23, 24. Three studies focussed on sexual HRQoL of patients with NMIBC and reported a high prevalence of sexual dysfunction in this patient cohort 21, 25, 26. The systematic review validates the present study and concluded that patients with NMIBC had worse physical, psychological and social QoL compared to the general population.

We acknowledge several limitations in our present study. The Brief‐IPQ is not validated in NMIBC unlike the EORTC‐QLQ NMIBC24 27. However, the Brief IPQ allows for rapid assessment of cognitive and emotional state of patients, which allows good comparison between other illness and cancers 17. Further, patients were interviewed ≥6 months after their NMIBC diagnosis; hence, their initial experience is dependent on their recall of their previous experience. In addition, 42.4% of patients did not complete the questionnaire; hence, this may introduce selection bias, although there was no difference in cancer grade, stage or risk (all *P* > 0.05) between patient who completed the questionnaire and those who did not.

Our results highlight that patients with NMIBC have a poor perception of disease control and believe that their disease will continue over a prolonged period of time. This is particularly more pertinent in the elderly. Patients are most psychologically affected during the interval between cancer diagnosis following cystoscopy and tumour resection at TURBT. Further, health awareness about the causes of bladder cancer remained poor with a significant number of patients unaware of the cause of bladder cancer. Our results suggest that this represents an area for future research. Psychological support and prompt TURBT following initial bladder cancer diagnosis would help to improve the mental health of patients with NMIBC.

## DETECT II collaborators

Participating centres and investigators (*principle investigators at each centre): W.S. Tan, P. Khetrapal, A. Sridhar, H. Baker, F. Ocampo, J.D. Kelly* (University College London Hospitals NHS Foundation Trust [UCLH]), N. Whotton, K. Dent, S. Pearson, J. Hatton, M. Newton, E. Heeney, K. Green, S. Evans, M. Rogers* (Northern Lincolnshire and Goole NHS Foundation Trust), A. Dann, J. Cook, M. Cornwell, R. Mills* (Norfolk and Norwich University Hospital), H. Knight, S. Maher, A. Rane* (East Surrey Hospital), S. Thomas, S. Reyner, G. Vallejera, P. Adeniran, S. Masood* (Medway Maritime Hospital), S. Ridgway, M. Coulding, H. Savill, J. Mccormick, M. Clark, G. Collins* (Tameside General Hospital), K. Jewers, S. Keith, G. Bowen, J. Hargreaves, K. Riley, S. Srirangam* (East Lancashire Hospitals NHS Trust), R. Mistry, J. Chadwick, S. Cocks, R. Hull, A. Loftus, L. Dawson*, (Royal Bolton Hospital), H. Roberts, C. Main, S. Jain* (St James’s University Hospital), C. Waymont, J. Rogers, A. Grant, V. Carter, H. Heap, C. Lomas, P. Cooke* (New Cross Hospital), Y. Baird, S. Moore, S. Greenslade, J. Margalef, I. Chadbourn, M. Harris, J. Hicks* (Western Sussex Hospitals NHS Foundation Trust), P. Clitheroe, S. Connolly, S. Hodgkinson, H. Haydock, A. Sinclair* (Stepping Hill Hospital), E. Storr, L. Cogley, S. Natale* (Derriford Hospital), W. Lovegrove, S. Smith, K. Smith* (King's Mill Hospital), D. Hewitt, R. Sriram* (University Hospitals Coventry), K. Atkinson, L. Royle, J. Madine, K. MacLean* (Royal Cornwall Hospital), J. Walsh, A.M. Guerdette, M. Hill, D. Payne* (Kettering General Hospital), A. Power, J. Cannon, L. Devereaux, A. Thompson* (Royal Albert Edward Infirmary), L. Scarratt, T. Hodgkiss, D. Johnstone, J. Johnson, J. Allsop, J. Rothwell, K. Connolly, J. Cherian* (The Pennine Acute Hospitals NHS Trust), H. Wardle, D. Wilson, A. Bayles* (University Hospital of North Tees), S. Pelluri, J. Pati* (Homerton Hospital), A. Gherman, C. Scott, S. Madaan* (Darent Valley Hospital), J.A. Taylor, E. Edmunds, J. Moore* (East Sussex Healthcare NHS Trust), A. Rees, S. Williams, S. Caddy, S. Dukes, A. Goffe* (Dorset County Hospital), K. Buckhorn, L. Nichols, P. Acher* (Southend Hospital), K. Baillie, K. Middleton, C. Proctor, J. Cresswell, A. Chilvers, M. Cain, A. Vaux, D. Watson* (James Cook University Hospital), S. Bradfield, H. Gregory, H. Mostafid* (Royal Surrey County Hospital), L. Kehoe, S. Drakeley, J.A. Davies, L. Williamson, R. Krishnan* (Kent and Canterbury Hospital), N. Lunt, P. Hill, H. Burns, B. Townley, L. Wilkinson, H. Wassall, A. Sinclair* (Macclesfield Hospital), J. Hunt, B. Holbrook, L. Stancombe, J. Morrison, L. Vankoutrik, S. Misra* (North Devon District Hospital), G. Fossey, A. Richards, K. Mcdonald, A. Henderson* (Maidstone Hospital), R. Fennelly, M. Tribbeck, K. Ames, J. Borwell* (Salisbury District Hospital), M. Kotze, K. Beesley, K. Rennie, T. Porter* (Yeovil District Hospital), A. Gipson, L. Piper, L. Bailey, A. Chrisopoulou, K. Slevin, F. McCartin, H. Warburton* (Wythenshawe Hospital), S. Hathaway‐Lees, K. Whetton, G. Delves* (Queen’s Hospital), A. Day, T. Bankole, S. Broadhead, S. Malde* (Guy's Hospital), M. Oblak, D. Ellis, S. Bishara, T. Barias‐Lara, I. Donkov* (West Middlesex University Hospital), H. Thatcher, H.M. Morris, C. Culmsee, A.H. Menzies, C. Bartlett, C. Cutting* (Royal Lancaster Infirmary), N. O'Brien, R. Jannapureddy, A. Kelkar* (Barking, Havering and Redbridge University Hospitals NHS Trust), J. Fitzgerald, S. Longhurst, C. Worth, A.M. Peracha* (Royal Derby Hospital), S. Mzazi, C. Poile, L. Griffiths* (Leicester Royal Infirmary), A. Cook, N. Barber, N. Brar, A. Alty, B. Zelhof, R. Blades* (Royal Preston Hospital).

## Funding

DETECT II was funded by the Medical Research Council (MRC). Additional funding was by the University College London Hospitals NHS Foundation Trust (UCLH) Biomedical Research Centre, The Urology Foundation and The Mason Medical Research Trust. It was supported both in the delivery and development by the UCL Surgical Interventional Trials Unit (SITU). NHS associated cost supported all standard of care investigations and procedures.

## Declaration of intent and financial disclosures

No direct or indirect commercial incentive is associated with publishing this article. The corresponding author certifies that, when applicable, a statement(s) has been included in the manuscript documenting Institutional Review Board (IRB), Ethics Committee or Ethical Review Board study approval; principles of Helsinki Declaration were followed in lieu of formal ethics committee approval; institutional animal care and use committee approval; all human subjects provided written informed consent with guarantees of confidentiality; IRB approved protocol number; animal approved project number.

## Ethical approval of studies and informed consent

DETECT II study protocol received Health Research Authority: London‐ Stanmore Research Ethics Committee approval on the 20th July 2016 and Health Research Authority approval on 30th August 2016 (Integrated Research Application System [IRAS] project ID: 203022, REC reference: 16/LO/1044). This trial is registered on clinicaltrials.gov NCT02781428.

## Supporting information


**Figure S1.** Flow chart.
**Table S1.** Patient interview outline.
**Table S2.** Brief Illness Perception Questionnaire scores.
**Table S3.** Patient demographics and tumour characteristics of patients interviewed.
