# Supplementary material for: Exploring patients’ experience and perception of being diagnosed with bladder cancer: a mixed‐methods approach
Source: BJU Int. 2020 Feb 12;125(5):669–78. doi: 10.1111/bju.15008 (PMC7318301; doi:10.1111/bju.15008)
Supplement: Supplementary file 1 — Figure S1. Flow chart. Table S1. Patient interview outline. Table S2. Brief Illness Perception Questionnaire scores. Table S3. Patient demographics and tumour characteristics of patients interviewed. [file BJU-125-669-s001.docx]

Figure S1: Flow chart.

**Total number of patients,**

**n= 370**

**Total number of patients who did not return questionnaire, n=157**

**Total number of questionnaires received, n= 213**

**Patients excluded from semi structured interviews:**

**Recurrent cancer, n=78**

**Not known, n =1**

**Patients with primary bladder cancer diagnosis, n= 134**

**Patients selective for qualitative analysis, n=20**

Table S1: Patient interview outline.

| **First thoughts when experiencing haematuria**  What have you been told by your doctors about bladder cancer?  Did your present with any symptoms which led to the diagnosis of bladder cancer  What do you understand about bladder cancer?  **Patients’ experience of being diagnosed with bladder cancer.**  When you were initially diagnosed with bladder cancer, how did you feel?  What were you worried of most?  **Subsequent experience with bladder cancer diagnosis**  How do you feel at the moment?  How long do you think you need to be monitored for your bladder cancer?  Has there been any consequence since your diagnosis of bladder cancer and has it affected your life in any way? |
| --- |

Table S2: Brief illness perception questionnaire scores

| **Component** | **New diagnosis**  **[n=135]**  **(mean ± SD)** | **Known bladder cancer**  **[n=78]**  **(mean ± SD)** | **P value** |
| --- | --- | --- | --- |
| 1. **Consequence (n=205)** | 2.8 ±2.5 | 3.0 ± 2.7 | 0.661 |
| 1. **Timeline (n=193)** | 5.3 ±3.5 | 7.5 ± 3.1 | <0.001 |
| 1. **Personal control (n-202)** | 3.3 ± 3.0 | 2.4 ± 3.3 | 0.046 |
| 1. **Treatment control (n-199)** | 8.2 ± 2.4 | 8.2 ± 2.8 | 0.942 |
| 1. **Identity (n=202)** | 2.6 ± 2.7 | 2.7 ± 2.8 | 0.695 |
| 1. **Concern (n-201)** | 5.7 ± 3.1 | 5.4 ± 3.6 | 0.593 |
| 1. **Coherence (n=204)** | 7.5 ± 2.6 | 8.0 ± 2.7 | 0.235 |
| 1. **Emotional representation (n=202)** | 4.0 ±3.0 | 3.4 ± 3.3 | 0.230 |
| **Overall score (n=184)** | 31.0 ± 12.4 | 34.0 ± 11.8 | 0.121 |

SD: standard deviation

Table S3: Patient demographics and tumor characteristics of patients interviewed.

| **Variable** | **N (%)** |
| --- | --- |
| **Age, median (IQR)** | 73.7 (61.8, 80.2) |
| **Gender, n (%)**  **Male** | 15 (75) |
| **Tumor grade, n (%)**  **G1**  **G2**  **G3** | 4 (20)  8 (40)  8 (40) |
| **Tumor stage, n (%)**  **pTa**  **pT1** | 15 (75)  5 (25) |
| **Disease risk, n (%)**  **Low**  **Intermediate**  **high** | 4 (20)  8 (40)  8 (40) |
| **Recent recurrence, n (%)**  **Yes** | 5 (25) |

IQR: interquartile range
